# Decreased expression of *Acsm2* disrupts energy metabolism and affects cell death in acute kidney injury

**DOI:** 10.3389/fcell.2026.1985288

**Published:** 2026-10-13

**Authors:** Binyu Wang, Yan Zha

**Affiliations:** 1 Medical College, Guizhou University, GuiYang, China; 2 Department of Nephrology, Guizhou Provincial People’s Hospital, Guiyang, China

**Keywords:** Acsm2, AKI, energy metabolism, programmed cell death, ScRNA-seq

## Abstract

**Background:**

Acute kidney injury (AKI) is a clinical syndrome characterized by high morbidity and a significant risk of mortality. Early diagnosis of AKI provides a crucial therapeutic window for treatment. There is an urgent need for universal biomarkers for the early diagnosis of AKI.

**Methods:**

We analyzed single-cell RNA sequencing (scRNA-seq) data of AKI and found that *Acsm2* was downregulated in the S1, S2, and S3 segments in AKI. Differential analysis, enrichment analysis, and pseudotime trajectory analysis were performed, in conjunction with animal experiments and transcriptome datasets, to explore the expression pattern and role of *Acsm2* in AKI.

**Results:**

Our study found that *Acsm2* was significantly downregulated in the S1, S2, and S3 segments of renal tubules during the early stages of AKI. *Acsm2* expression was correlated with key metabolic pathways, including fatty acid metabolism and mitochondrial function. The downregulation of *Acsm2* in AKI was associated with disrupted energy metabolism and increased cell death, including apoptosis, necroptosis, and pyroptosis in proximal tubular cells (PTCs).

**Conclusion:**

Downregulation of *Acsm2* in AKI is correlated with impaired energy metabolism and cell death in PTCs, suggesting that *Acsm2* may serve as a potential biomarker for the early diagnosis and intervention of AKI. Further studies on its mechanistic role could provide new insights into the pathophysiology of AKI and offer novel therapeutic strategies.

## 1 Introduction

Acute kidney injury (AKI) is a clinical syndrome characterized by high morbidity and a significant risk of mortality. The prevalence of AKI is approximately 10%–15% among hospitalized patients, while it exceeds 50% among those in intensive care units (ICUs) (Ronco et al., 2019). Globally, over 10 million people are diagnosed with AKI each year, and more than 1.7 million people die from AKI annually (Cui et al., 2024). Early diagnosis of AKI provides a crucial therapeutic window for treatment. However, current clinical tests based on creatinine levels and urine output are ineffective for the early diagnosis of AKI. There is an urgent need for universal biomarkers for the early diagnosis of AKI (Xiao et al., 2022). The main pathological manifestations of AKI are inflammation, vascular dysfunction, and injury and death of proximal tubular cells (PTCs). Among these, cell death is a major contributor to the progression of AKI (Linkermann et al., 2014). However, its underlying mechanisms remain incompletely understood and require further investigation.

In AKI, cell death occurs in various forms, including apoptosis, necroptosis, and pyroptosis (Zhao et al., 2023; Guerrero-Mauvecin et al., 2023; Li et al., 2024; Deng et al., 2021). Apoptosis, a form of programmed cell death, has been extensively studied in multiple AKI models and involves caspases, sphingomyelinases, Bcl-2-like proteins, and mitochondria (Linkermann et al., 2014; Zamzami and Kroemer, 2001; Gulbins et al., 2003). Necroptosis is mediated by the RIPK3-MLKL pathway, leading to plasma membrane rupture accompanied by inflammation and cytokine release, which has been widely investigated in ischemia-induced AKI models (Belavgeni et al., 2020; Maremonti et al., 2022; Wang F. et al., 2022). Pyroptosis is closely associated with the activation of the NLRP3 inflammasome, where NLRP3 activation triggers downstream inflammatory cytokines, contributing to the progression of AKI (Li et al., 2022a). These cell death pathways are closely linked to mitochondria-mediated energy metabolism (Zhao et al., 2023).

Energy metabolism disorders have been widely recognized as a major cause of cell death (Eckert et al., 2003; Deng et al., 2024; Varga et al., 2015; Mansouri et al., 2018), with energy insufficiency being one of the key contributing factors (Baker et al., 2024; L et al., 2024). Among the various metabolic pathways that sustain cellular energy, fatty acid metabolism is a primary energy source for PTCs. When fatty acid metabolism is disrupted, unmetabolized lipids and intermediate metabolites, such as ceramides and long-chain acyl-CoAs, accumulate (Kihara, 2016; Enkler et al., 2023; Yan et al., 2015). These metabolites inhibit the activity of oxidative phosphorylation complexes, reduce ATP production (Su et al., 2020; Lin et al., 2018; Burgin et al., 2023; Nsiah-Sefaa and McKenzie, 2016), and cause energy depletion, ultimately resulting in apoptosis (Hosseini et al., 2021; Gómez, 2023). In various types of AKI, fatty acid metabolism is significantly downregulated, driving energy depletion-induced cell death and worsening kidney injury (Zhao et al., 2023; Bhargava and Schnellmann, 2017; Wang B. et al., 2022; Xu et al., 2022).

The acyl-CoA synthetase (ACS) family catalyzes the ATP-dependent conversion of free fatty acids into acyl-CoA. Acyl-CoA undergoes β-oxidation in the mitochondria to generate acetyl-CoA, NADH, and FADH2. Acetyl-CoA subsequently enters the tricarboxylic acid (TCA) cycle, while NADH and FADH2 are fed into the electron transport chain (ETC) to drive ATP synthesis (Adeva-Andany et al., 2019; Ki et al., 2002). The Acyl-CoA synthetase (ACS) family includes several subfamilies, such as short-chain (ACSS), medium-chain (ACSM), long-chain (ACSL), and very long-chain (ACSVL) synthetases. The Acyl-CoA synthetase medium-chain family member 2 (*Acsm2*) gene is highly conserved across species. In the human genome, the *ACSM2A* and *ACSM2B* genes share significant homology with *Acsm2*. *Acsm2* is suggested to participate in fatty acid metabolism and the glycine conjugation pathway, as well as contribute to the development and maturation of proximal tubular cells. However, its precise role and function require further investigation (Watanabe et al., 2020).

In this study, we analyzed single-cell RNA sequencing (scRNA-seq) data from mouse models of cisplatin-induced AKI and IRI-induced AKI, finding that *Acsm2* plays an important role in both models. Through differential analysis, enrichment analysis, and correlation analysis, we discovered that *Acsm2* is associated with fatty acid metabolism and energy metabolism. Furthermore, the expression level of *Acsm2* is negatively correlated with apoptosis, necroptosis, and pyroptosis.

## 2 Materials and methods

### 2.1 Ethical approval

The animal study was reviewed and approved by the Animal Experimental Ethical Inspection Form of Guizhou Medical University (approval no. 2305127).

### 2.2 Mice with induced AKI

C57BL/6 male mice (8 weeks old) were purchased from Chongqing Tengxin Biotechnology Co., Ltd. The mice were housed under specific pathogen-free (SPF) conditions and allowed to acclimate for 1 week. They were then randomly divided into four groups (3 mice per group). Cisplatin (25 mg/kg) or saline were administered via intraperitoneal injection. Blood and kidney samples were collected at 24, 48 and 72 h post-injection for further analysis.

### 2.3 Histopathology

The kidney tissues were sectioned into 4 μm thick paraffin sections and stained using an HE staining kit (Beijing Solarbio Science and Technology Co. Ltd., Beijing, China), following the manufacturer’s instructions.

### 2.4 Kidney function

Serum creatinine and blood urea nitrogen were measured according to the instructions provided by the assay kits (Nanjing Jiancheng Bioengineering Institute, China).

### 2.5 Quantitative real-time PCR

Renal RNA was extracted using Trizol (Thermo Fisher, United States of America) reagent, followed by reverse transcription into single-stranded cDNA using reverse transcription reagent (Thermo Fisher, United States of America). RNA levels of *Acsm2* were examined by qPCR mix (TIANGEN, China), and a S1000 PCR Thermal cycler was used to detect the system. Relative gene expression was calculated using 2−^ΔΔCT^ with GAPDH as the endogenous control gene. The following primer sequences were used: *Acsm2* (forward: 5′-TGG​GGG​AAT​GAG​ATT​TCC​TGC-3′, reverse: 5′-CTT​CAC​TCA​GTT​CTC​GGA​AGC-3′); GAPDH (forward: 5′- AGG​TCG​GTG​TGA​ACG​GAT​TTG -3′, reverse: 5′-GGG​GTC​GTT​GAT​GGC​AAC​A-3′).

### 2.6 Dataset sources

The scRNA-seq data used in this study were obtained from the GSE197266 and GSE139107 datasets on the GEO platform, and the human acute kidney injury (AKI) transcriptome datasets were obtained from the GSE37838, GSE30718, and GSE139061 datasets. From GSE197266, we selected scRNA-seq data of mouse kidneys under control, cisplatin treatment, and ischemia–reperfusion injury conditions; GSE139107 was downloaded together with the original cell annotations provided by the dataset authors. GSE37838 and GSE30718 were both profiled on the GPL570 [HG-U133_Plus_2] Affymetrix Human Genome U133 Plus 2.0 Array: GSE37838 comprised 70 renal allograft implantation biopsy samples from 53 deceased donors, whereas GSE30718 contained 28 indication biopsy samples from transplants with AKI and 11 pristine protocol biopsy samples from stable transplants. GSE139061 was generated on the GPL20301 Illumina HiSeq 4000 platform (*Homo sapiens*), comprising 39 native human renal biopsy samples with AKI and 9 reference nephrectomy samples.

### 2.7 scRNA-seq data pretreatment

We used the Seurat R package (version 5.1.0) for the analysis of the scRNA-seq dataset GSE197266. Using the subset function, we isolated cells from control mice, cisplatin-treated mice, and mice subjected to ischemia-reperfusion injury. Low-quality cells were filtered out based on gene counts below 400 or above 4,000, as well as cells with mitochondrial transcription levels exceeding 50%. This relatively high threshold was adopted because renal tubular epithelial cells require abundant mitochondria to support high-energy-demanding active-transport processes (Tang et al., 2023). A total of 31,763 cells were retained for further analysis. We identified 2,000 highly variable genes for SCT integration, and the clustering results were visualized using UMAP. We downloaded the GSE139107 dataset along with the metadata uploaded by the authors, which contains their annotations for the dataset. We used the AddMetaData function to incorporate the metadata information.

### 2.8 Cell type identification

We subsequently analyzed by principal component analysis (PCA) using the RunPCA function for highly variable genes. We used the Harmony package (version 1.2.0) to eliminate batch-related biases. Cells were clustered using the FindClusters function, followed by UMAP analysis. Different types of cells were identified based on the expression levels of known canonical markers within each cluster using Seurat’s FindAllMarkers function.

### 2.9 Differentially expressed genes (DEGs) and gene functional enrichment analysis

The DEG analysis for PTCs was conducted by first using the subset function to extract PTCs, followed by the Seurat FindMarkers function to analyze differential expression between the control (CON) and AKI (CP, IRI) groups. The criteria were set at |avg_log2FC| > 0.25, min. pct >0.25, and an adjusted *P*-value <0.05. Differentially expressed genes were visualized using the ggplot2 R package. The openxlsx R package (version 4.2.5.2) was used to generate circular heatmaps illustrating gene expression across different groups. Cells were divided into high- and low-expression groups based on the median expression of *Acsm2*, and DEG analysis was performed using the same criteria. Visualization was carried out with the EnhancedVolcano function. Gene Ontology (GO) and Kyoto Encyclopedia of Genes and Genomes (KEGG) analyses were performed using the clusterProfiler R package (version 4.10.1) for genes enriched with an adjusted *P*-value <0.05. The results were visualized with ggplot2 (v3.5.0).

### 2.10 Analysis of murine *Acsm2* expression in scRNA-seq dataset GSE139107

*Acsm2* expression was analyzed with the published snRNA-seq dataset for mouse kidneys with ischemic reperfusion injury (GSE139107) on R package Seurat (version 5.1.0). The results were visualized with DotPlot ggplot2 (version 3.5.0).

### 2.11 Pseudotime analysis

Pseudotemporal analysis was performed using Monocle2 (version 2.30.1). We converted Seurat object to CDS object. We defined the branch-dependent genes by branched expression analysis modeling (BEAM) function in Monocle2 and then cataloged them into three clusters in a pseudotime manner. Finally, we performed GO and KEGG analysis of each gene cluster.

### 2.12 Gene set activity score, correlation analysis, and ROC curve analysis

The specific gene sets used for both scRNA-seq data and transcriptome data were downloaded from the Molecular Signatures Database (MSigDB, https://www.gsea-msigdb.org/gsea/msigdb/). AUCell (version 1.26.0) was performed to assess enrichment scores for specific gene sets in scRNA-seq data, and the Wilcoxon test was used to evaluate the statistical differences between high and low *Acsm2* expression groups. The Gene Set Variation Analysis (GSVA) algorithm was used to infer the specific pathway scores for all AKI samples in the transcriptome data. Then, we calculated the correlation between *Acsm2* expression and specific pathway activity scores using Spearman’s correlation analysis. In addition, receiver operating characteristic (ROC) curve analysis was conducted using the pROC package (version 1.18.5) in R to evaluate the diagnostic performance of human *ACSM2A* and *ACSM2B* for AKI based on the GSE30718 and GSE139061 datasets.

### 2.13 Statistical analysis

The data were analyzed using GraphPad Prism software (version 8.0.1). Normally distributed data are presented as mean ± SEM. Comparisons among multiple groups were performed using one-way analysis of variance (ANOVA). *P* < 0.05 was considered significant.

## 3 Results

### 3.1 Single-cell profiling of the kidney landscape under AKI conditions induced separately by CP and IRI

To analyze the renal transcriptional characteristics of AKI, we selected scRNA-seq data from normal mice (Control), cisplatin (CP), and ischemia-reperfusion-injury (IRI) from the GSE197266 dataset, encompassing a total of 31,763 cells, to analyze transcriptional profiles of kidneys in AKI models. Thirteen cell types were identified based on well-established canonical markers (Figures 1A,B, Supplementary Figure S1D, Supplementary Tables S1–S3), and we examined the changes in the relative proportions of these cell types after CP induction and IRI induction (Figures 1C,D, Supplementary Figure S1E, Supplementary Table S1). Supplementary Table S1 provides the canonical marker genes and cell-type proportion statistics for each of the thirteen cell types. Among these, the relative percentage of PT, DCT, and LOH cells significantly decreased, while the proportions of macrophages, neutrophils, and endothelial cells significantly increased. In addition to the significant changes in cell type proportions, the gene expression profiles (upregulated and downregulated genes) were also markedly altered. The AKI groups (CP, IRI) showed significant changes in the gene expression profiles of these 13 cell types (Figures 1E,F, Supplementary Tables S1, S6). Injury to PTCs is one of the key features of AKI (Lee et al., 2024), and PTCs exhibited significant DEGs. CP induction led to upregulation of 1,088 genes and downregulation of 502 genes in PTCs, while IRI induction resulted in upregulation of 154 genes and downregulation of 602 genes. Therefore, we focused our follow-up analysis on PTCs.

**FIGURE 1:**
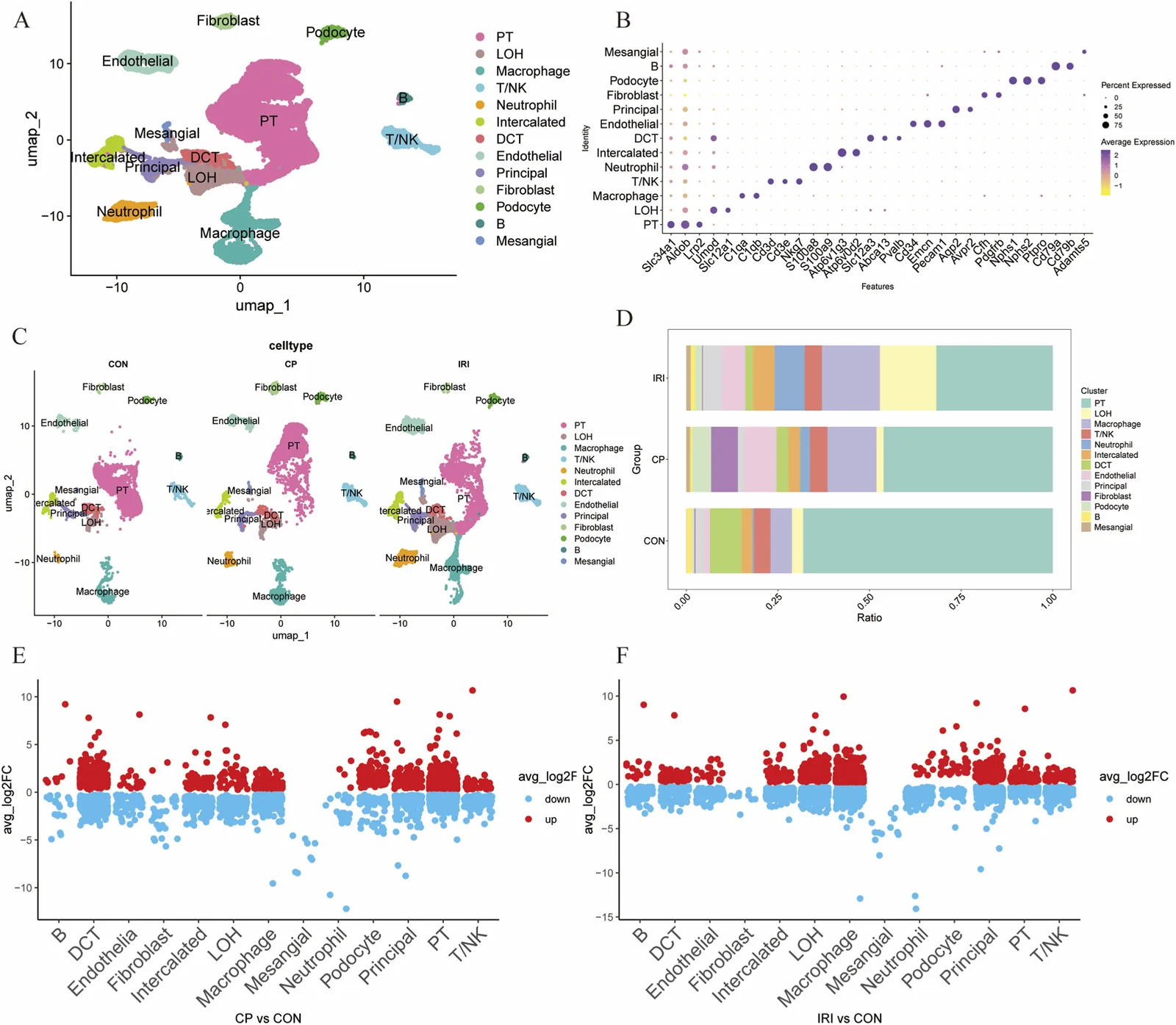
Single-cell RNA sequencing (scRNA-seq) profiling of renal tissues from mouse models of AKI induced separately by CP and IRI **(A)** Uniform manifold approximation and projection (UMAP) displays 13 distinct cell clusters. **(B)** Dotplot shows the expression of marker genes for each cell type. **(C)** UMAP plots for the control, CP and IRI group. **(D)** Cluster distribution in each group. **(E)** Distributions of up- and downregulated genes for each cell type after CP induction. **(F)** Distributions of up- and downregulated genes for each cell type after IRI. PT: proximal renal tubular cells; LOH: loop of Henle cells; T/NK: T lymphocytes/natural killer cells; DCT: distal convoluted tubule cells; EC: endothelial cells; B cell: B lymphocytes; CON: control; CP: cisplatin; IRI: ischemia-reperfusion injury.

### 3.2 Single-cell analysis of renal tubular landscapes under AKI conditions induced separately by CP and IRI

To examine the transcriptional characteristics of PTCs in AKI, we defined four cell types (Figures 2A,B, Supplementary Tables S4–S6) and assessed the changes in the relative proportions of these cell types across different groups (Figures 2C,D, Supplementary Table S6). Compared to the control group, the proportion of S1 cells decreased, while the proportion of S2 cells increased in the AKI groups, likely because S1 cells have the highest mitochondrial density and metabolic activity, making them more susceptible to damage (Chrysopoulou and Rinschen, 2024). Additionally, following IRI induction, the relative proportion of proliferative PTCs significantly increased, whereas no significant increase in proliferative PTCs was observed after CP induction.

**FIGURE 2:**
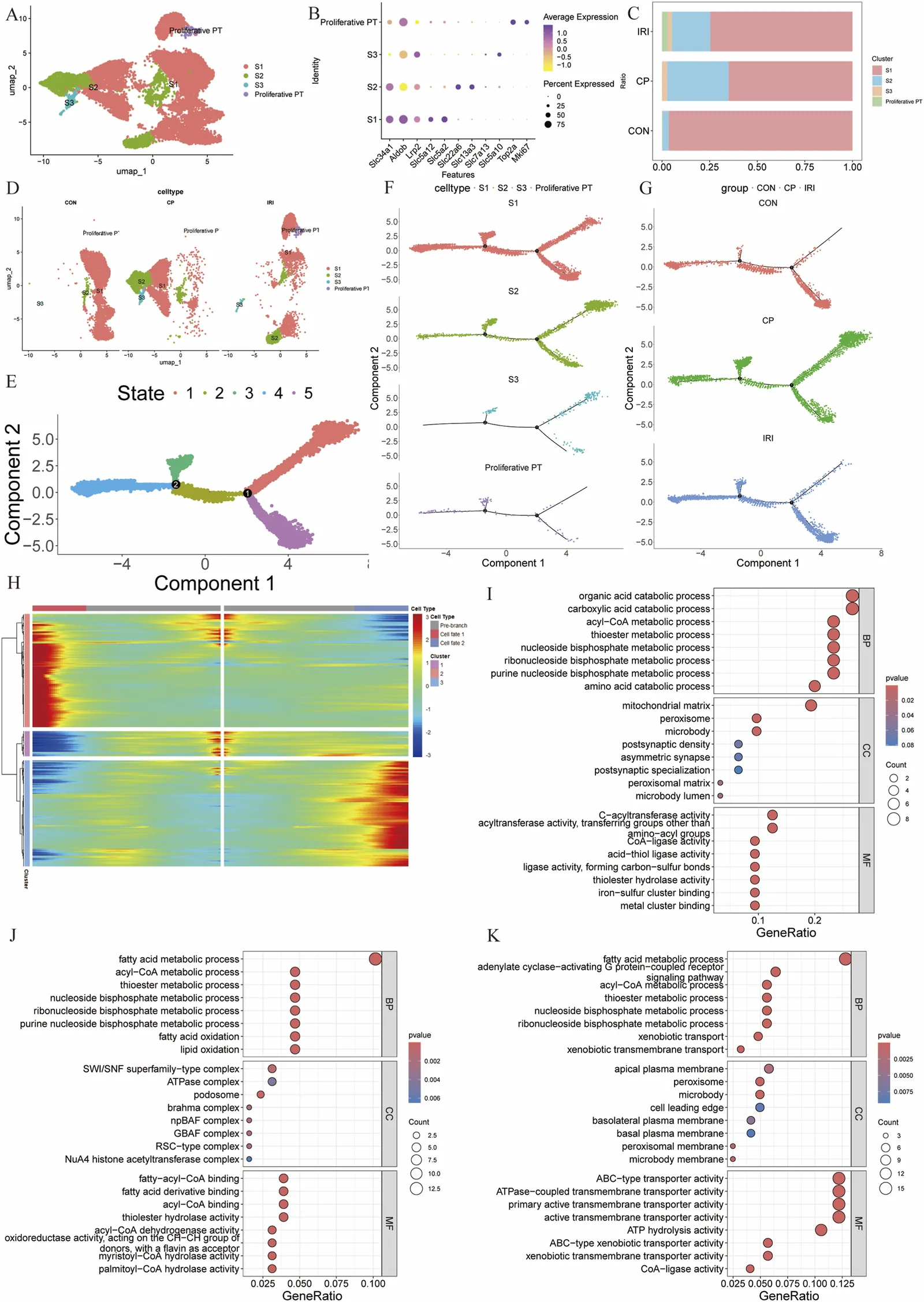
Single-cell RNA sequencing (scRNA-seq) profiling of PTCs from mouse models of AKI induced separately by CP and IRI. **(A)** Uniform manifold approximation and projection (UMAP) displays 4 distinct cell clusters. **(B)** Dotplot shows the expression of marker genes for each cell type. **(C)** Cluster distribution in each group. **(D)** UMAP plots for the S1, S2, S3 and Proliferative PT. **(E)** Pseudotime trajectory analysis of PTCs. **(F)** Different celltypes on the pseudotime trajectory. **(G)** Different groups on the pseudotime trajectory. **(H)** BEAM heatmap analysis identified genes associated with cellfate1 and cellfate2 differentiation. **(I)** Gene Ontology (GO) enrichment analysis of cluster1 in the BEAM heatmap. **(J)** GO enrichment analysis of cluster2 in the BEAM heatmap. **(K)** GO enrichment analysis of cluster3 in the BEAM heatmap. CON: control; CP: cisplatin; IRI: ischemia-reperfusion injury.

To infer the dynamic changes of PTCs under AKI conditions, we performed pseudotime trajectory analysis, which divided the cells into five states (Figure 2E, Supplementary Figure S2A). In the control group, most cells (primarily S1, S2, and S3) differentiated from state 2 to state 5 at node 1. In contrast, under AKI conditions, the number of cells progressing toward state 1 significantly increased (Figures 2F,G, Supplementary Figure S2B,C). To identify the genes underlying these changes, BEAM analysis was conducted at the bifurcation point of node 1 (Figure 2H, Supplementary Table S7), and the three clusters in the BEAM heatmap represented cellfate1 (state 1), cellfate2 (state 5), and the intermediate node. Genes in Cluster 1 were mainly associated with fate 1 and were suppressed in this cluster; these genes are primarily involved in organic acid catabolism process, carboxylic acid catabolic process, and acyl-CoA metabolic process (Figure 2I, Supplementary Table S8). Cluster 2 was primarily related to fatty acid metabolic process, acyl-CoA metabolic process, and thioester metabolic process, processes that were upregulated in the fate1 trajectory and downregulated in the fate2 trajectory (Figure 2J, Supplementary Table S8). Cluster 3 was associated with fate2, with processes like fatty acid metabolic process, and adenylate cyclase-activating G protein-coupled receptor signaling pathway being activated in this cluster (Figure 2K, Supplementary Table S8).

### 3.3 *Acsm2* is significantly downregulated in AKI

To investigate gene changes in AKI, we analyzed DEGs in S1, S2, and S3 (Figure 3A, Supplementary Figure S3, Supplementary Table S9). A total of 340 common DEGs were identified in S1, 502 in S2, and 7 in S3 under CP-induced AKI and IRI-induced AKI (Figure 3B), with *Acsm2* being downregulated in S1, S2, and S3 (Figure 3C). Pseudotime analysis using Monocle2 showed that *Acsm2* expression progressively decreased along the state 1 trajectory (Figure 3D).

**FIGURE 3:**
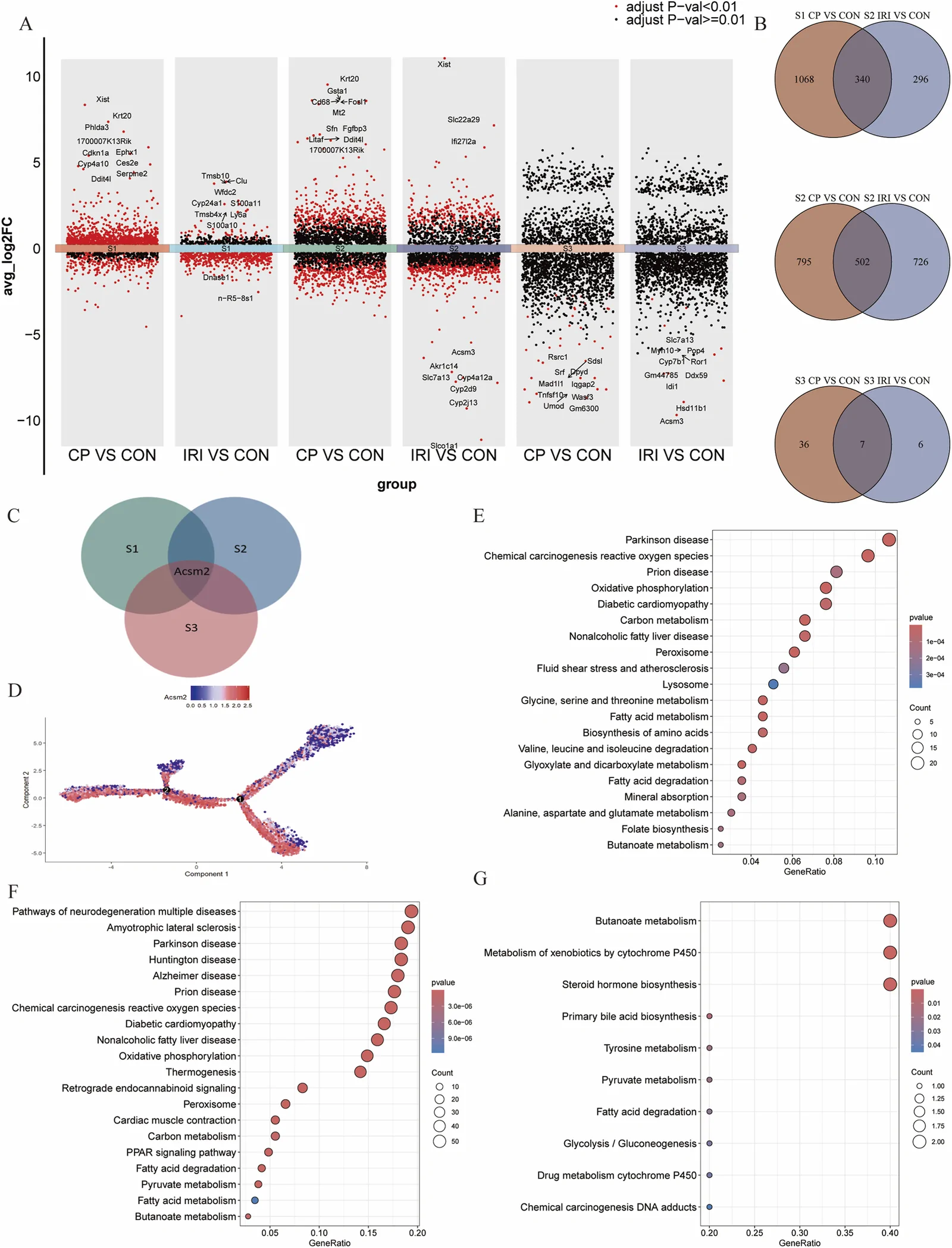
Downregulation of *Acsm2* across tubular S1, S2, and S3 segments in AKI. **(A)** Differential gene expression analysis showing up- and downregulated genes in the AKI (CP, IRI) groups compared to the CON group across the S1, S2, and S3 clusters. An adjusted *p*-value <0.01 is indicated in red, while an adjusted *p*-value ≥0.01 is indicated in black. **(B)** The Venn diagram shows the shared differentially expressed genes (DEGs) between the AKI (CP, IRI) groups and the CON group across the S1, S2, and S3 clusters. **(C)** The Venn diagram shows that *Acsm2* differs among the S1, S2, and S3 clusters. **(D)** *Acsm2* expression dynamics on the pseudotime trajectories. **(E)** Kyoto Encyclopedia of Genes and Genomes (KEGG) enrichment analysis of shared DEGs in the S1 cluster. **(F)** KEGG enrichment analysis of shared DEGs in the S2 cluster. **(G)** KEGG enrichment analysis of shared DEGs in the S3 cluster. AKI: acute kidney injury; CP: cisplatin; IRI: ischemia-reperfusion injury; CON: control.

We performed GO and KEGG enrichment analysis on these common DEGs (Supplementary Table S8). Both S1 and S2 were significantly enriched in pathways related to fatty acid metabolism and fatty acid degradation (Figures 3E,F). Although fewer common DEGs were found in S3, they were still enriched in fatty acid degradation pathways (Figure 3G). Given that acyl-CoA is a key intermediate in fatty acid metabolism, we hypothesize that *Acsm2* may be associated with the disrupted fatty acid metabolism observed in AKI.

### 3.4 Expression Pattern of *Acsm2* in AKI

To investigate *Acsm2* gene expression at different time points in the IRI model, we analyzed its expression patterns in the entire kidney and in various clusters from the published snRNA-seq dataset of mouse kidneys with ischemic reperfusion injury (GSE139107) (Kirita et al., 2020). In mouse IRI, *Acsm2* was downregulated at early time points (4 and 12 h) but was re-expressed 48 h after injury, with expression gradually increasing (Figure 4A). Notably, before IRI (0 h), *Acsm2* was primarily expressed in the proximal renal tubules, with lower expression levels in S3 compared to S1 and S2. However, after IRI at 4, 12, and 48 h, the expression of *Acsm2* expanded to the whole kidney (Figure 4B, Supplementary Figure S4). Currently, there is limited research on *Acsm2* in CP-induced AKI. To investigate the temporal expression changes of *Acsm2* in CP-induced AKI, we established a time-gradient model for CP-induced AKI. The results showed a significant increase in kidney weight/body weight 72 h after CP injection (Figure 4C). HE staining revealed progressively worsening renal damage over time (Figure 4D). Serum creatinine and blood urea nitrogen levels significantly increased 72 h after injection, confirming the successful establishment of the AKI model (Figures 4E,F). qPCR results indicated that *Acsm2* expression did not significantly change at 24 h, likely due to the time required for cisplatin-induced tubular epithelial cell injury and death to manifest. Over time, *Acsm2* expression gradually decreased from 48 h to 72 h (Figure 4G).

**FIGURE 4:**
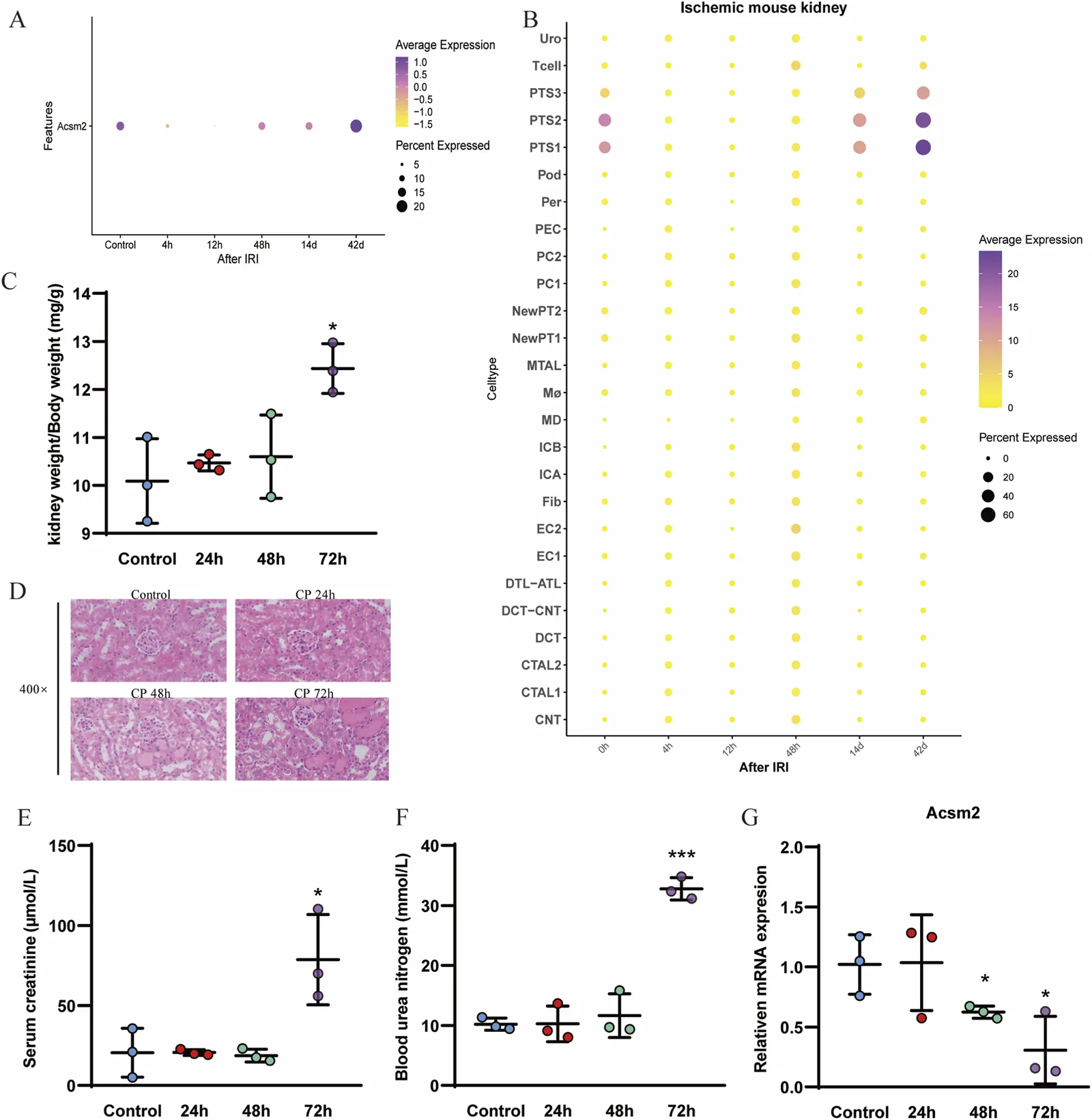
Expression Pattern of *Acsm2* in AKI. **(A)** *Acsm2* expression in the whole kidneys at different time points after IRI. **(B)** *Acsm2* expression in different clusters at different time points after IRI. **(C)** Kidney weight/body weight at different time points after CP induction. **(D)** HE staining of kidneys at different time points after CP induction. **(E)** Serum creatinine levels at different time points after CP induction. **(F)** Blood urea nitrogen levels at different time points after CP induction. **(G)** The qPCR results of *Acsm2* at different time points after CP induction. CP: cisplatin; IRI: ischemia-reperfusion injury. ∗*P* < 0.05. ∗∗∗*P* < 0.001.

Considering the functional conservation of *Acsm2* across species, we further evaluated the expression and diagnostic performance of its human homologs *ACSM2A* and *ACSM2B* in AKI using two independent human datasets (GSE30718 and GSE139061). Both *ACSM2A* and *ACSM2B* were significantly downregulated in AKI samples in these two datasets (*P* < 0.05). ROC analysis showed that both genes achieved AUC values greater than 0.7, supporting their potential value as novel diagnostic biomarkers for AKI (Supplementary Figure S5).

### 3.5 *Acsm2*-related genes are significantly enriched in energy metabolism pathways

To explore the mechanism of *Acsm2*, we categorized the cells from the GSE197266 dataset into high and low *Acsm2* expression groups based on *Acsm2* expression levels and performed differential expression analysis (Figures 5A,B, Supplementary Table S10). We performed GO enrichment analysis on the differentially expressed genes (Figures 5C,D, Supplementary Table S8). In terms of biological processes (BP), the upregulated genes were significantly enriched in the generation of precursor metabolites and energy, fatty acid metabolic process, and acyl-CoA metabolic process. The downregulated genes were mainly enriched in processes such as RNA splicing, actin filament organization, mRNA processing, and regulation of actin filament-based processes. Regarding cellular components (CC), the upregulated genes were primarily enriched in the mitochondrial matrix, oxidoreductase complex, and mitochondrial respirasome. The downregulated genes were enriched in the cell leading edge, spliceosomal complex, and cytoskeleton-related components. In the molecular function (MF) category, the upregulated genes were closely associated with oxidoreductase activity, and NAD/NADP binding. The downregulated genes were linked to RNA polymerase II-specific DNA-binding transcription factor binding and cytoskeleton binding. KEGG enrichment analysis indicated that upregulated genes were significantly enriched in key metabolic pathways, including oxidative phosphorylation, citrate cycle (TCA cycle), and pyruvate metabolism. The downregulated genes were significantly enriched in pathways related to apoptosis and cellular senescence (Figures 5E,F). AUCell package analysis revealed that the high-expression group had significantly higher scores in fatty acid metabolism, oxidative phosphorylation and TCA cycle, suggesting enhanced pathway activity (Figure 5G, Supplementary Tables S11,S12).

**FIGURE 5:**
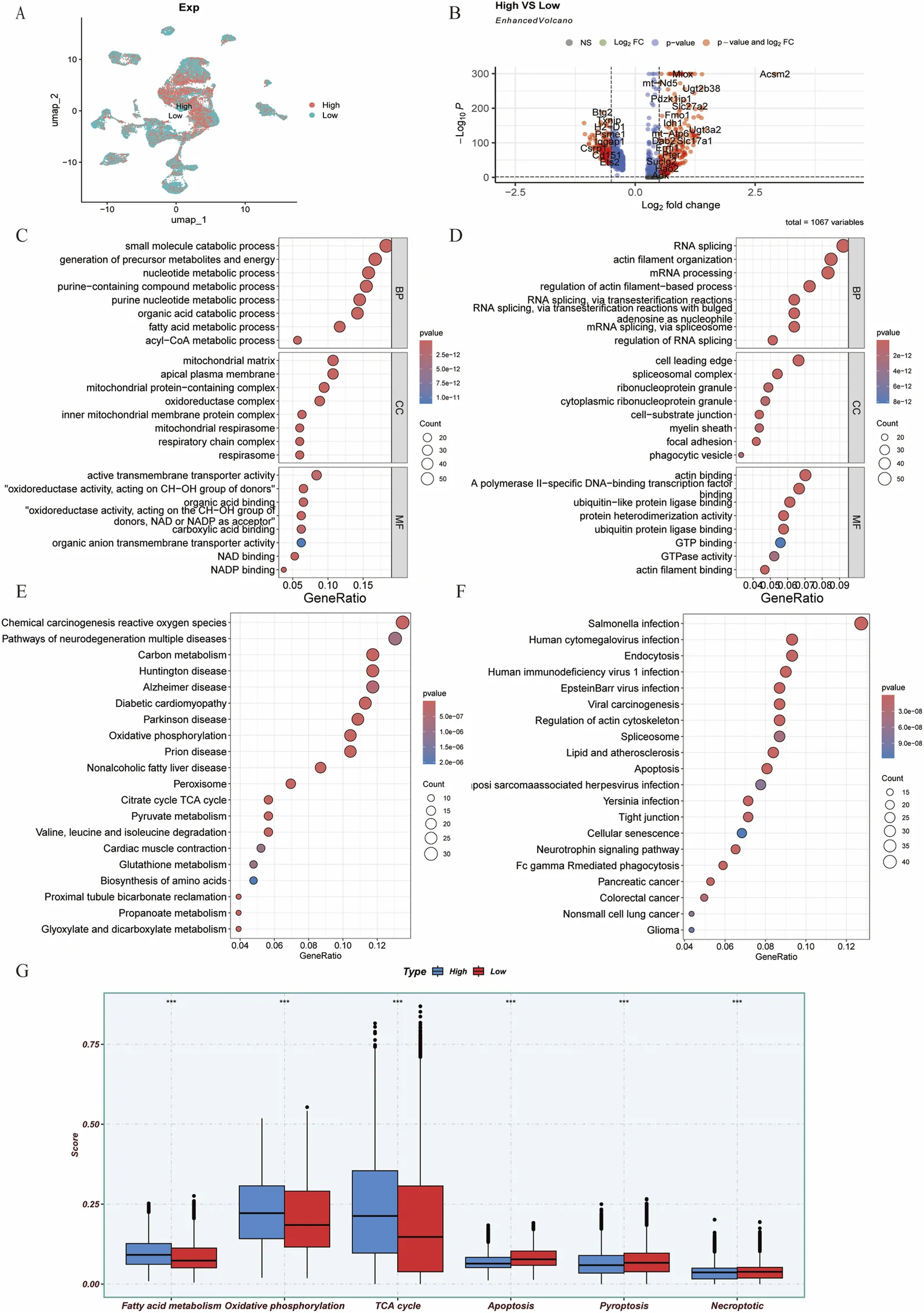
Pathway activity characteristics of *Acsm2* high- and low-expression PTCs in AKI. **(A)** Uniform manifold approximation and projection (UMAP) plot displays the *Acsm2* high expression group and *Acsm2* low expression group. **(B)** Volcano plot shows the differentially expressed genes (DEGs) between the *Acsm2* high expression group and the *Acsm2* low expression group. **(C)** Gene Ontology (GO) enrichment analysis of upregulated genes **(D)** GO enrichment analysis of downregulated genes **(E)** Kyoto Encyclopedia of Genes and Genomes (KEGG) analysis of upregulated genes. **(F)** KEGG analysis of downregulated genes. **(G)** Scores for specific pathway activities evaluated by the AUCell algorithm. ∗∗∗*P* < 0.001. High: High *Acsm2* expression group; Low: Low *Acsm2* expression group.

Given the limited functional studies on the homologous genes *ACSM2A* and *ACSM2B* in the human genome, we performed gene set variation analysis (GSVA) on energy metabolism pathways in the GSE37838 dataset (Supplementary Figure S6, Supplementary Table S13) and examined their correlation with *ACSM2A* and *ACSM2B*. In fatty acid metabolism, both *ACSM2A* (R = 0.68) and *ACSM2B* (R = 0.68) exhibited strong positive correlations (Figures 6A,B). Regarding oxidative phosphorylation, *ACSM2A* (R = 0.46) and *ACSM2B* (R = 0.41) showed positive correlations (Figures 6C,D). In the TCA cycle, both *ACSM2A* (R = 0.48) and *ACSM2B* (R = 0.44) also exhibited strong positive correlations, suggesting that *ACSM2A* and *ACSM2B* are associated with the maintenance of mitochondrial function and energy metabolism (Figures 6E,F).

**FIGURE 6:**
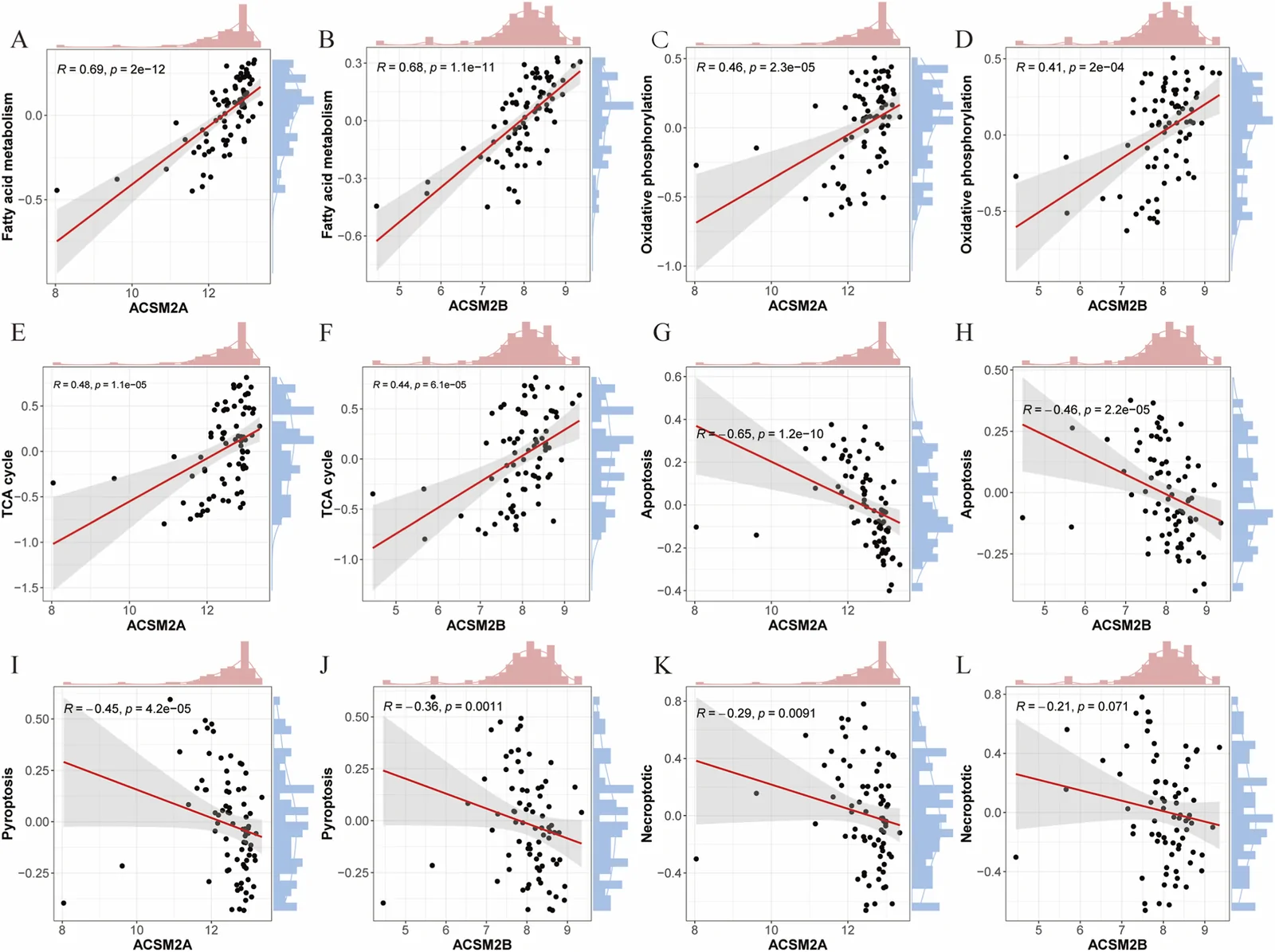
Correlation of *ACSM2A* and *ACSM2B* expression with energy metabolism and programmed cell death pathways in human AKI. A-L Correlation of *ACSM2A* or *ACSM2B* with specific pathways. **(A)** *ACSM2A* and fatty acid metabolism. **(B)** *ACSM2B* and fatty acid metabolism. **(C)** *ACSM2A* and oxidative phosphorylation. **(D)** *ACSM2B* and oxidative phosphorylation. **(E)** *ACSM2A* and TCA cycle. **(F)** *ACSM2B* and TCA cycle. **(G)** *ACSM2A* and apoptosis. **(H)** *ACSM2B* and apoptosis. **(I)** *ACSM2A* and pyroptosis. **(J)** *ACSM2B* and pyroptosis. **(K)** *ACSM2A* and necroptotic. **(L)** *ACSM2B* and necroptotic.

In summary, high expression of *Acsm2* is significantly enriched in energy metabolism pathways, including fatty acid metabolism and mitochondrial function. The low expression of *Acsm2* in AKI may correlate with reduced energy production and disrupted energy metabolism in PTCs.

### 3.6 The expression of *Acsm2* is closely associated with cell death

Given that energy metabolism imbalance and energy depletion are important factors contributing to PTC death in AKI, we used the AUCell package to score the activity of cell death pathways in high and low *Acsm2* expression groups. Our results indicated that *Acsm2* expression is closely related to various forms of cell death. Scores for apoptosis, necroptosis, and pyroptosis were significantly higher in the low expression group (Figure 5G, Supplementary Tables S11,S12), suggesting that reduced *Acsm2* expression correlates with higher activity of these programmed cell death pathways, which is accompanied by increased PTC death.

Similarly, *ACSM2A* was significantly negatively correlated with multiple programmed cell death pathways, whereas ACSM2B showed a similar trend but did not reach statistical significance for necroptosis (Supplementary Table S13). *ACSM2A* (R = −0.46) and *ACSM2B* (R = −0.46) were negatively correlated with apoptosis (Figures 6G,H). *ACSM2A* (R = −0.45) and *ACSM2B* (R = −0.36, p = 0.0011) were negatively correlated with pyroptosis (Figures 6I,J). *ACSM2A* (R = −0.29) was also negatively correlated with necroptosis, although no such association was observed for *ACSM2B* (Figures 6K,L).

In summary, low *Acsm2* expression correlates with elevated activity of apoptosis, necroptosis, and pyroptosis. The expression patterns of *ACSM2A* and *ACSM2B* are largely consistent with the findings of mouse *Acsm2*, whereas *ACSM2B* shows no significant correlation with necroptosis.

## 4 Discussion

AKI is a condition of acute renal dysfunction caused by multiple etiologies, including ischemia, toxicity, drug reactions, infections, and other factors (Turgut et al., 2023; Darmon et al., 2017). Due to the complex etiology and diverse clinical manifestations of AKI, traditional diagnostic methods often have limitations. Serum creatinine, blood urea nitrogen, and urine output are commonly used diagnostic criteria (Yoon et al., 2022; Gobe et al., 2015). However, these indicators typically only show alterations after significant decline in renal function, making it difficult to provide timely diagnostic information during the early stages of the disease (Wen and Parikh, 2021). This complicates the early diagnosis of AKI, highlighting the urgent need for the development of more sensitive and universal biomarkers to enable early identification of the disease (Xiao et al., 2022).

This study, through analysis of the single-cell datasetGSE197266, found that the expression of *Acsm2* was downregulated in the S1, S2, and S3 segments of renal tubules during the early stages of AKI. This result aligns with previous findings from the R Ariel Gomez team, who reported a downregulation of *Acsm2* in both AKI and CKD. Other studies have shown that proteomics analysis of acute kidney injury biomarkers in a rat exertional heat stroke model indicates downregulation of *Acsm2* (Wen et al., 2023). *ACSM2A* is also significantly downregulated in AKI (Zhang Y. et al., 2022). Specifically, *Acsm2* showed significant downregulation at 48 h post-IRI or 72 h post-CP. Notably, *Acsm2* exhibited consistent downregulation across the S1, S2, and S3 segments of renal tubules in AKI induced by various etiologies. Compared with other known biomarkers, downregulation of *Acsm2* demonstrates broader applicability. As such, *Acsm2* is a promising candidate biomarker for early identification and intervention of AKI.

*Acsm2* belongs to the ACS family (Watanabe et al., 2020; Zhang F. et al., 2022), which catalyzes the ATP-dependent conversion of free fatty acids into acyl-CoA, a substrate that undergoes β-oxidation in the mitochondria to generate energy, subsequently entering the TCA cycle and the electron transport chain to drive ATP synthesis (Houten and Wanders, 2010)^.^ It has been suggested that *Acsm2* may be involved in fatty acid metabolism and glycine conjugation pathways (van der S and luis, 2018). Previous studies have indicated that *Acsm2* is predominantly expressed in the kidney, with relatively lower expression in the liver, and its expression pattern resembles that of PTCs in terms of function and maturation (Watanabe et al., 2020). Our study shows that the high expression group of *Acsm2* in AKI is significantly enriched in pathways related to fatty acid metabolism, oxidative phosphorylation, and the TCA cycle, all of which provide energy to PTCs. Therefore, we hypothesize that downregulation of *Acsm2* in AKI leads to a reduction in energy supply to PTCs, thereby driving cell death. Recent studies have also shown that *ACSM2A* is closely related to mitochondrial function and is downregulated in hepatocellular carcinoma (Ma et al., 2025), which provides theoretical support for our research.

Our results suggest that downregulation of *Acsm2* in AKI may promote apoptosis, necroptosis, and pyroptosis in PTCs. Its homologs, *ACSM2A* and *ACSM2B*, exhibited negative correlations with apoptosis and pyroptosis in AKI. *ACSM2A* was additionally negatively correlated with necroptosis, while such significant association was not detected for *ACSM2B*.Previous studies have shown that *ACSM2A* expression is positively correlated with the glomerular filtration rate (Ledo et al., 2015). Ningning Li’s team found that melatonin upregulates *Acsm2* expression while reducing apoptosis in PTCs (Li et al., 2022b). Treatment with Tongxinluo (TXL) upregulated *ACSM2B* expression and reduced apoptosis in cardiac microvascular endothelial cells (CMECs) (Li et al., 2017). Our findings suggest that the downregulation of *Acsm2* may be an important contributing factor in AKI. However, whether the downregulation of *Acsm2* in AKI leads to insufficient energy supply, thereby causing cell death, remains to be validated.

Several limitations of the present study should be acknowledged. First, the identification and characterization of the *Acsm2*-associated cell death pathway were based entirely on computational and transcriptomic analyses of publicly available datasets. We did not directly manipulate *Acsm2* expression (e.g., through *in vitro* overexpression or knockout) to clarify its causal role in AKI. Second, all transcriptomic data were derived from public GEO datasets. Although standard normalization and integration procedures were applied, the influence of inter-study batch effects, differences in sequencing platforms, and variable cohort compositions cannot be completely excluded. Third, the *in vivo* validation in the present study served as an exploratory preliminary assessment with three animals per group. Although this meets the statistical requirements for preliminary studies, the sample size remains limited, and these findings will need to be confirmed in larger cohorts. Finally, although the downregulation of *ACSM2A*/*ACSM2B* was confirmed in independent human AKI transcriptome cohorts, these findings have not yet been validated in independent human renal tissue samples by experimental approaches such as immunohistochemistry, quantitative PCR, or Western blotting. Future studies employing *in vitro* and *in vivo* gain- and loss-of-function models, together with independent human tissue validation, are warranted to further elucidate the functional role of *Acsm2* in AKI.

In summary, downregulation of *Acsm2* in AKI is a key factor contributing to the reduced activity of pathways such as fatty acid metabolism, oxidative phosphorylation, and the TCA cycle. This study also reveals a potential link between *Acsm2* and cell death, although whether cell death is primarily driven by a reduction in energy supply remains to be further investigated. Currently, studies on the specific mechanisms of *Acsm2* in AKI are limited, and exploring its mechanisms in AKI may provide new strategies for the early diagnosis and treatment of AKI.

## Data Availability

The original contributions presented in the study are included in the article/Supplementary Material, further inquiries can be directed to the corresponding author.

## Supplementary material

SUPPLEMENTARY MATERIAL FIGURE S1 Quality control and single-cell analysis of GSE197266. **(A)** The number of genes for each cell for Three groups after scRNA-seq data quality control. **(B)** The number of transcripts for each cell for three groups after scRNA-seq data quality control. **(C)** The percentage of mitochondrial genes for each cell for three groups after scRNA-seq data quality control. **(D)** UMAP plot of the expression of marker genes for each cell type. **(E)** Bar graph illustrating the ratio of various cell types in mouse models of acute kidney injury (AKI) induced by cisplatin (CP) and ischemia-reperfusion injury (IRI).

SUPPLEMENTARY MATERIAL FIGURE S2 Pseudotime trajectory analysis of PTCs. **(A)** Different states on the pseudotime trajectory. **(B)** Different celltypes on the pseudotime trajectory. **(C)** Different groups on the pseudotime trajectory.

SUPPLEMENTARY MATERIAL FIGURE S3 Differential gene expression analysis in the AKI (CP, IRI) groups compared to the CON group. **(A)** Volcano Plot of Differential Gene Analysis between CP group and CON group in S1. **(B)** Volcano Plot of Differential Gene Analysis between IRI group and CON group in S1. **(C)** Volcano Plot of Differential Gene Analysis between CP group and CON group in S2. **(D)** Volcano Plot of Differential Gene Analysis between IRI group and CON group in S2. **(E)** Volcano Plot of Differential Gene Analysis between CP group and CON group in S3. **(F)** Volcano Plot of Differential Gene Analysis between IRI group and CON group in S3. CON: control; CP: cisplatin; IRI: ischemia-reperfusion injury.

SUPPLEMENTARY MATERIAL FIGURE S4 A-F UMAP displays distinct cell clusters at different time points after IRI-induced AKI. **(A)** 0h. **(B)** 4h. **(C)** 12h. **(D)** 48h. **(E)** 14d. **(F)** 42d.G-L UMAP plot shows the expression levels of Acsm2 at different time points after IRI-induced AKI. **(G)** 0h. **(H)** 4h. **(I)** 12h. **(J)** 48h. **(K)** 14d. **(L)** 42d. UMAP: Uniform manifold approximation and projection; IRI: ischemia-reperfusion injury.

SUPPLEMENTARY MATERIAL FIGURE S5
**(A,D)** Box plots showing the expression levels of ACSM2A and ACSM2B in control and AKI samples from the GSE30718 **(A)** and GSE139061 **(D)** datasets. Both genes were significantly downregulated in AKI samples. **(B,C,E,F)** Receiver operating characteristic (ROC) curve analysis evaluating the diagnostic performance of ACSM2A **(B,E)** and ACSM2B **(C,F)** in the GSE30718 **(B,C)** and GSE139061 **(E,F)** datasets.

SUPPLEMENTARY MATERIAL FIGURE S6
**(A)** Boxplots of gene expression levels in GSE37838 before normalization (left) and after normalization (right). **(B)** Heatmap showing the expression levels of ACSM2A and ACSM2B. **(C)** Heatmap of GSVA activity scores for specific pathways. GSVA; Gene Set Variation Analysis.

SUPPLEMENTARY MATERIAL TABLE S1 Cell Type Expression and Proportional Data in Kidney.

SUPPLEMENTARY MATERIAL TABLE S2 Differentially expressed genes in each cell cluster used for cell type annotation in the kidney.

SUPPLEMENTARY MATERIAL TABLE S3 Differentially expressed genes in each celltypes in the kidney.

SUPPLEMENTARY MATERIAL TABLE S4 Differentially expressed genes in each cell cluster used for cell type annotation in the PTCs.

SUPPLEMENTARY MATERIAL TABLE S5 Differentially expressed genes in each celltypes in the PTCs.

SUPPLEMENTARY MATERIAL TABLE S6 Cell Type Expression and Proportional Data in PTCs.

SUPPLEMENTARY MATERIAL TABLE S7 Genes associated with cell differentiation in BEAM analysis of PTCs.

SUPPLEMENTARY MATERIAL TABLE S8 GO and KEGG Enrichment Analysis.

SUPPLEMENTARY MATERIAL TABLE S9 Differentially expressed genes in the AKI groups (cisplatin and ischemia-reperfusion injury) compared with the control group.

SUPPLEMENTARY MATERIAL TABLE S10 Differentially expressed genes between high and low expression groups of Acsm2.

SUPPLEMENTARY MATERIAL TABLE S11 Pathway-related Gene Set.

SUPPLEMENTARY MATERIAL TABLE S12 Scores for specific pathway activities evaluated by the AUCell algorithm.

SUPPLEMENTARY MATERIAL TABLE S13 Correlation between Acsm2 expression levels and GSVA activity scores of various pathways.
